# Exploring extracellular vesicles in zoonotic helminth biology: implications for diagnosis, therapeutic and delivery

**DOI:** 10.3389/fcimb.2024.1424838

**Published:** 2024-08-06

**Authors:** Abdul Qadeer, Abdul Wajid, Hafiz Abdul Rafey, Saqib Nawaz, Sawar Khan, Sajid Ur Rahman, Khalid J. Alzahrani, Muhammad Zahoor Khan, Mohammad Nafi Solaiman Alsabi, Hanif Ullah, Sher Zaman Safi, Zanxian Xia, Muhammad Zahoor

**Affiliations:** ^1^ Department of Cell Biology, School of Life Sciences, Central South University, Changsha, China; ^2^ Shanghai Veterinary Research Institute, Chinese Academy of Agricultural Sciences, Shanghai, China; ^3^ Faculty of Pharmacy, Gomal University, Dera Ismail Khan, Pakistan; ^4^ Shifa College of Pharmaceutical Sciences, Faculty of Pharmaceutical and Allied Health Sciences, Shifa Tameer-e-Millat University, Islamabad, Pakistan; ^5^ Institute of Molecular Biology and Biotechnology, The University of Lahore, Lahore, Pakistan; ^6^ Department of Food Science and Engineering, School of Agriculture and Biology, Shanghai Jiao Tong University, Shanghai, China; ^7^ Department of Clinical Laboratories Sciences, College of Applied Medical Sciences, Taif University, Taif, Saudi Arabia; ^8^ College of Agricultural Science and Engineering, Liaocheng University, Liaocheng, Shandong, China; ^9^ Department of Basic Veterinary Medical Sciences, Faculty of Veterinary Medicine, Jordan University of Science and Technology, Irbid, Jordan; ^10^ West China School of Nursing/West China Hospital, Sichuan University, Chengdu, China; ^11^ Faculty of Medicine, Bioscience and Nursing, MAHSA University, Jenjarom, Selangor, Malaysia; ^12^ Department of Molecular Medicine, Institute of Basic Medical Sciences, University of Oslo, Oslo, Norway

**Keywords:** extracellular vesicles, helminth, miRNA, immunomodulation, host-parasite interaction

## Abstract

Extracellular vesicles (EVs) have emerged as key intercellular communication and pathogenesis mediators. Parasitic organisms’ helminths, cause widespread infections with significant health impacts worldwide. Recent research has shed light on the role of EVs in the lifecycle, immune evasion, and disease progression of these parasitic organisms. These tiny membrane-bound organelles including microvesicles and exosomes, facilitate the transfer of proteins, lipids, mRNAs, and microRNAs between cells. EVs have been isolated from various bodily fluids, offering a potential diagnostic and therapeutic avenue for combating infectious agents. According to recent research, EVs from helminths hold great promise in the diagnosis of parasitic infections due to their specificity, early detection capabilities, accessibility, and the potential for staging and monitoring infections, promote intercellular communication, and are a viable therapeutic tool for the treatment of infectious agents. Exploring host-parasite interactions has identified promising new targets for diagnostic, therapy, and vaccine development against helminths. This literature review delves into EVS’s origin, nature, biogenesis, and composition in these parasitic organisms. It also highlights the proteins and miRNAs involved in EV release, providing a comprehensive summary of the latest findings on the significance of EVs in the biology of helminths, promising targets for therapeutic and diagnostic biomarkers.

## 1 Introduction

Parasitic diseases transmitted from animals to humans are common. Infection can occur through contaminated food, water, soil, or animal contact. Despite causing significant global health issues and substantial financial losses in the livestock industry, parasitic zoonosis is often overlooked (Torgerson and Macpherson, 2011). Human population growth and socioeconomic shift drive migration into new ecological regions and change animal husbandry practices, affecting disease emergence and burden. Enhanced diagnostics are uncovering that many zoonoses have a higher burden than previously understood (Macpherson, 2005). Additionally, new syndromes are being attributed to practice zoonoses, further adding to the disease burden. Climate change may alter the transmission dynamics of parasitic zoonosis in endemic areas and enable some parasites to spread to previously unaffected regions (Torgerson and Macpherson, 2011). Monitoring water quality has become crucial for comprehending the environmental conditions affecting humans, animals, and vegetation, in line with the One Health approach (Orusa et al., 2020; Orusa et al., 2023; Orusa et al., 2024). The connection between human and animal health and environmental conditions is vital for effectively managing and conserving global biodiversity (Viani et al., 2024). In recent years, it has become clearer that personal behaviors significantly impact exposure to zoonotic agents, often due to globalization and the convenience of international travel. Zoonotic diseases account for a major share (60.3%) of new infectious diseases. Among 1500 pathogens that can infect humans, 66 are protozoa and 287 are helminths, highlighting a considerable disease burden and shifts in how these diseases are distributed (Alebie and Tewachew, 2021).

Parasitic helminths, commonly known as worms, have a major impact on society, infecting more than 1.5 billion people globally and imposing additional burdens on livestock systems. This affects animal welfare, food security, and sustainable agriculture. These pathogens can remain in their natural hosts for years, largely because they can manipulate the host’s immune response and physiological state (White et al., 2023). Parasitic helminths fall into two main phyla: Platyhelminthes, which includes trematodes and cestodes, and Nematoda, which comprises nematodes like Soil-transmitted helminths (STHs), filarial worms (causing diseases like onchocerciasis and lymphatic filariasis), and schistosomes are the primary organisms causing diseases in humans and animals (Ullah et al., 2022). The significant economic impact of helminth infections, coupled with the rise of drug resistance and the absence of effective vaccines, highlights the urgent need for worldwide attention and the development of innovative control strategies, including exploration into potential treatments and vaccines (Sánchez-López et al., 2021). Despite their unique life cycles and tissue tropism preferences, all helminths establish persistent infections by modulating the host’s immune response to enhance their survival chances (Ryan et al., 2020). This manipulation primarily involves the secretion of molecules at the interface between the parasites and the host, collectively referred to as excretory/secretory products (ESP). ESP consists of various elements such as proteins, lipids, metabolites, nucleic acids, and extracellular vesicles (EVs), all contributing to the complex interplay between the parasite and its host (Maizels et al., 2018). Parasites such as helminths have been shown to produce and release EVs, which can be internalized by host cells and influence the host immune response (Coakley et al., 2015; Mekonnen et al., 2018). EVs also contribute to the pathology of parasitic diseases and offer significant potential as innovative diagnostic tools and therapeutic agents against a variety of parasitic pathogens (Marcilla et al., 2014). EVs are small membrane-surrounding structures secreted by a wide range of cell types, containing rich cargo that is important in cell-cell communication, and attracted wide attention due to their unique functional relevance in host-parasite interaction (Mu et al., 2020). This review described the nucleic acid, proteomic, and lipid composition of EVs from helminths, with a focus on their ability as vaccine candidates, delivery material, and diagnostic biomarkers within the EVs.

### 1.1 What are extracellular vesicles?

Extracellular vesicles (EVs) also referred to as “platelet dust” were 1^st^ time observed 50 years ago in plasma by Wolf (Hessvik and Llorente, 2018). These EVs are shed by a wide range of cell types. Initially, they were considered akin to “cellular garbage cans” responsible for discarding cellular material deemed unnecessary (Thébaud and Stewart, 2012). However, comprehensive research on EVs has uncovered their diverse origin, characteristics, and nature, leading to the use of various terms to describe them. These terms cover various aspects such as scale (using prefixes like micro or nano: such as microparticles, microvesicles (MVs), nano-vesicles, nanoparticles), their origin within cells or tissue (like prostasomes, oncosomes), suggested roles (such as calcifying matrix vesicles, autosomes, tolerosomes), or their existence outside cells (with prefixes exo or ecto, including ectosomes, exosomes, exovesicles, exosome-like vesicles) (Colombo et al., 2014). Exosomes, having a diameter smaller than 150 nm, predominantly appear in three distinct forms determined by their size and method of release: primarily manifest in three forms based on their release mechanism and size: microvesicles, shedding particles, and apoptotic bodies exceeding 100 nm in size (Hessvik and Llorente, 2018). This review will not delve deeper into other categories of vesicles that originate directly from the cell’s plasma membrane, whether from living or decaying cells.

A wide spectrum of organisms, spanning from eukaryotes like amoebae, *Caenorhabditis elegans*, parasites, and mammals to prokaryotic cells, have shown the ability to discharge vesicles into the extracellular environment (Colombo et al., 2014). These EVs comprise a diverse set of membranous vesicles that arise from either the endosome or the plasma membrane, playing a vital role in intercellular communication. Their involvement in different physiological and pathological functions varies based on size and cellular origin, and they are present in numerous biological fluids like milk, blood, urine, and saliva (Qadeer et al., 2021).

### 1.2 Discovery and origin of exosome

In the 1980s, exosomes were initially termed endosomal-derived vesicles secreted by reticulocytes (Colombo et al., 2014). Exosomes are small EVs (usually 30 to 150 nanometers in diameter) that originate in the endosomal pathway. They are identified by markers such as CD63, CD81, and TSG101. The broader term “Extracellular vesicles” was later coined to encompass membrane vesicles containing cytoplasm from secreting cells, that are encapsulated in a lipid bilayer (Skotland et al., 2020). Researcher indicates that the mechanism of EV secretion from cells has likely remained relatively consistent throughout its evolution (Raposo and Stoorvogel, 2013). In most of the studies on EV secretion, cultured cells have been used to investigate the endosomal origin and biogenetic of these secreted vesicles. The main types of EV actively produced by living cells are exosomes, which are 30–100 nm and originate from the endosomal pathway of eukaryotic cells (Park et al., 2023). Most cultured cells have been observed to release both microparticles (MPs) and MV-derived endosomes (Camussi et al., 2010). Microvesicles are bigger EVs (varying in size from 100 to 1,000 nanometers) that are shed directly from the plasma membrane of cells. These vesicles may transport a wide range of materials, including proteins, lipids, and nucleic acids. According to the MISEV recommendations, microvesicles should be separated from exosomes based on their biosynthesis and size range (Camussi et al., 2010). In addition to exosomes, and microvesicles, there may be other populations of larger EVs that have distinct biogenesis and functional characteristics. However, the precise mechanisms underlying the assembly and sorting of exosomes are not fully understood, mainly because a universal sorting single applicable to all cell types has not been identified yet (Camussi et al., 2010; Garcia-Martin et al., 2022).

### 1.3 Helminths parasite-derived extracellular vesicles

Parasitic diseases pose a significant global health burden, affecting billions of people. Among the nearly 400 parasitic species known to infect humans, around 90 of them lead to substantial clinical morbidities and mortalities (Marcilla et al., 2014). Unfortunately, these diseases are often neglected due to their prevalence in impoverished regions. To gain insight into the mechanisms of parasitic infections, researchers have extensively studied the secretion of EV by various unicellular and multicellular parasites. Trematodes like *Echinostoma caproni*, *Fasciola hepatica*, *Dicrocoelium dendriticum* (Marcilla et al., 2014), *Schistosoma japonicum* (Wang et al., 2015), *Schistosoma mansoni* (Marcilla et al., 2014; Wang et al., 2015), and *Opisthorchis viverrini* have been utilized as parasitic models for EV research (Chaiyadet et al., 2015).

Several studies have indicated the role of EVs produced by parasites or parasitized cells in the development of parasitic infections (Marcilla et al., 2014). EVs with pathogenic attributes can function as signaling agents in both parasite-to-parasite communications between parasites and their hosts. This phenomenon enables parasites to uphold their typical parasitic functions, contributing to the development of host pathogenesis (Marcilla et al., 2012; Barteneva et al., 2013). Parasitic EVs have also been implicated in parasite infection maintenance, pathogenicity, migration inside host tissue (Cwiklinski et al., 2015), and immune regulation (Buck et al., 2014). In the beginning, Marcilla and his colleagues demonstrated that exosome-like vesicles released by *E. Caproni* and *F. hepatica* were internalized by host intestinal cells, implying the involvement of these vesicles in facilitating communication between the parasite and their host. The parasite’s extracellular vesicles are taken up by the cells most likely by endocytosis, which confirmed interspecific communication medicated by exocytic vesicles (Marcilla et al., 2012). In another study, the soluble and vesicular secretome of *F. hepatica* were juxtaposed, outlining the impact of EVs on processes such as biogenesis, cargo sorting, membrane trafficking, cytoskeleton control, and modulation of the immune response during the progression of the parasitic infection (Fromm et al., 2015). In a previous study concerning the glycocalyx of *S. mansoni* cercariae structures resembling MVBs were identified near the tegument of flatworm schistosomula (Samuelson and Caulfield, 1985). Exosomes like vesicles, are secreted by adult schistosomes and can be internalized by mammalian cells. These vesicles carry miRNA payloads that hold the potential to modulate the expression of genes within the host (Wang et al., 2015; Zhu L. et al., 2016). Similarly, research focused on *S. mansoni* has revealed that these schistosomes are capable of releasing EVs enriched with small non-coding RNAs and proteins, some of which share similarities with vaccine candidates (Nowacki et al., 2015; Sotillo et al., 2016).

Parasitic EVs also hold potential significance in orchestrating the host immunological responses to foster tolerance towards the parasitic presence within the final host (Riaz and Cheng, 2017). EVs originating from the nematode parasite *Brugia malayi* have been identified in the bloodstream of hosts, suggesting their potential influence at distinct sites beyond the immediate host microenvironment (Zamanian et al., 2015). Recent research into helminth extracellular vesicles has markedly improved our comprehension of their interactions with hosts during infection. While strides have been made in understanding the functions of these vesicles in helminth development and disease progression, there remains considerable room for further exploration. Enhanced insight into their roles could potentially unveil new biomarkers and therapeutic interventions for neglected tropical diseases (Riaz and Cheng, 2017).

A significant portion of understanding regarding extracellular vesicle (EVs) formation in helminths is derived from research conducted on the liver fluke, *F. hepatica* (Cwiklinski et al., 2015). This parasite gives rise to at least two distinct subpopulations of EVs, which exhibit variations in size, cargo composition, and likely sites of origin, as elucidated by a comprehensive proteomic analysis of its secretome (Bennett et al., 2020). Within one of these subpopulations of smaller EVs (with diameters ranging from 30–100 nm), proteins such as Hsp70, ALIX, tetraspanin CD63, and various Rab GTPases have been identified. These findings suggest that *F. hepatica* indeed releases exosomes. By employing mass spectrometry, it was determined that these smaller secreted vesicles originated from endosomal compartments. Notably up to 10 proteins associated with endosomal sorting complexes required for the transports (ESCRT) pathway were uncovered, showing their association with these vesicles (Cwiklinski et al., 2015). Furthermore, the vesicles contained ESCRT-associated proteins ALIX and syntenin, alongside members of the ESCRT-III complex and the Vps4 ATPase. These components play critical roles in the processes of the intraluminal vesicle (ILV) budding and vesicle abscission thus shedding light on the mechanism underpinning EV formation (Cwiklinski et al., 2015).

Exosomes derived from *F. hepatica* possess essential membrane structuring proteins, encompassing various tetraspanins, annexins, an acid sphingomyelinase, a phospholipase B2-like enzyme, and cholesterol transporters Niemann Pick C1 and C2 (Cwiklinski et al., 2015). These membrane structures, also known as detergent-resistant microdomains, or TEMs, possess distinct biophysical characteristics (de Gassart et al., 2003). In the context of *F. hepatica*, and potentially other helminths, the exosomal membranes exhibit elevated rigidity under neutral pH conditions. This characteristic potentially contributes to maintaining structural integrity and persistence within host bile and other bodily fluids (Simbari et al., 2016). For instance, exosome membranes from the nematode parasite *H. polygyrus* showcase enrichment in plasminogens and specialized ether phospholipids. This enrichment impacts greater stiffness of the vesicle compared to the human exosomes (Simbari et al., 2016). As a result, it can be speculated that the distinctive composition of lipids and proteins in parasite EVs membrane has evolved to ensure optimal protection of the encapsulated cargo within the vesicle lumen (de la Torre-Escudero et al., 2016).

## 2 Composition of exosomes

**Figure 1:**
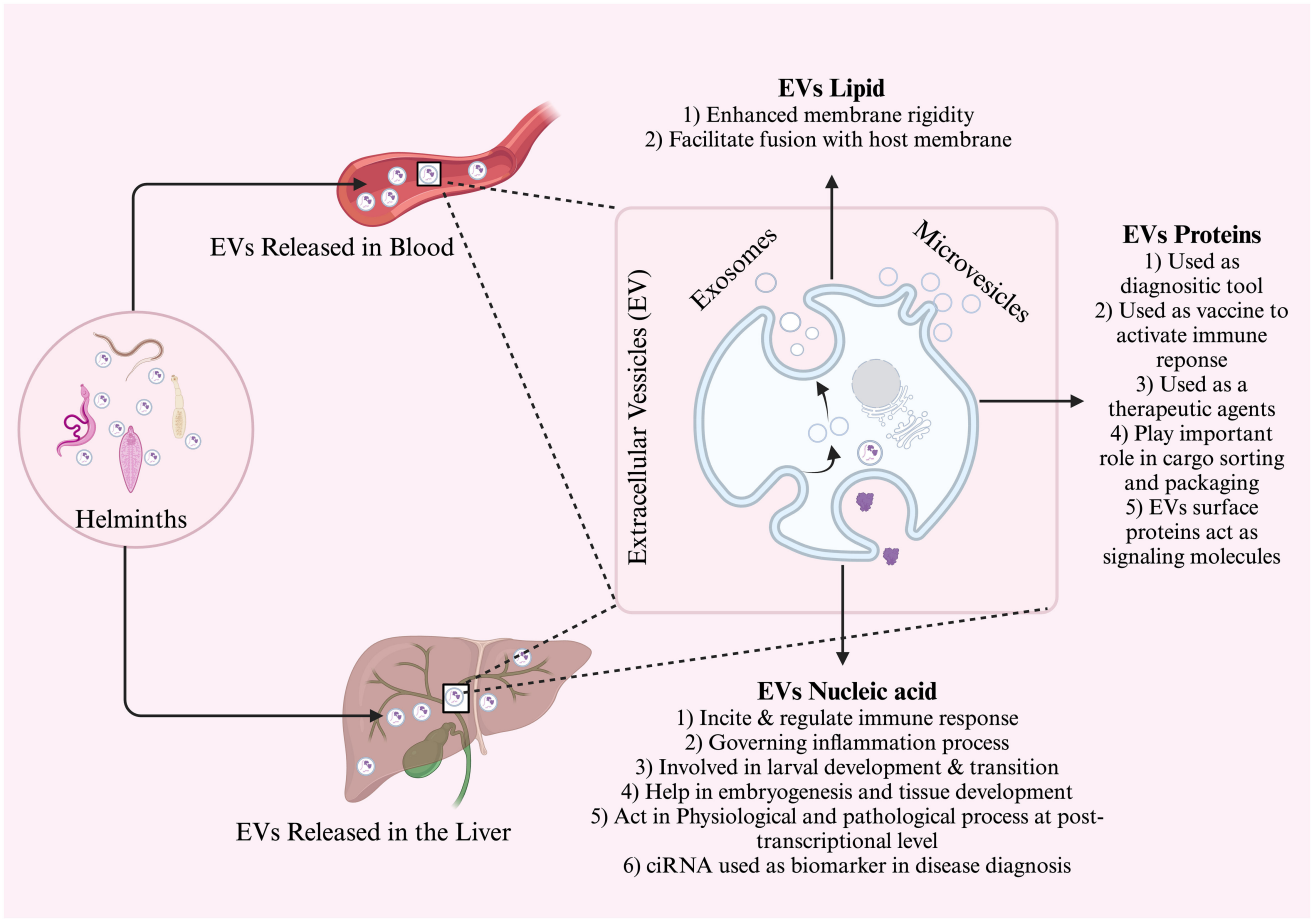
General schematic diagram of extracellular vesicle discharge in the bloodstream and liver. The EVs are composed of lipids, proteins, and nucleic acid. Each of these components has a different function and is used mostly in intracellular communication, diagnosis, and prognostic biomarkers.

EVs encompass a diverse range of biologically active proteins, lipids, and nucleic acids (**Figure 1**), which possess the potential to transmit and regulate signals to recipient cells (Qadeer et al., 2021). In this overview, we present the present status of the literature and pinpoint instances where endeavors have been carried out or are ongoing to delve into the molecular constituents of distinct EV subtypes. Initially, techniques relying on antibodies for protein detection, such as western blotting, and immuno-EM, were used to characterize the proteins present in extracellular vesicles (EVs). However, in the 1990s, the advent of proteomic analysis methods greatly facilitated the identification of previously unknown proteins in EV samples on a large scale (Colombo et al., 2014). Early proteomic studies revealed distinct sets of cellular proteins in exosomes, with some variations depending on the cell type that secretes them (Colombo et al., 2014).

While proteins from the nucleus, mitochondria, endoplasmic reticulum, and Golgi complex were frequently detected, proteins from the Endosome, Plasma membrane, and cytosol were generally not found in exosomes. These findings underscore the selectiveness of vesicle formation, suggesting that exosomes constitute a distinct subcellular compartment rather than a random assortment of cellular fragments (Théry et al., 2001; Théry et al., 2009; Chaput and Théry, 2011). Proteomic analysis of specific EVs revealed a significant overlap in protein expression between exosomes and vesicles derived from the plasma membrane. This implies that there may still be undiscovered proteins unique to exosomes found in plasma membrane-derived vesicles. Furthermore, some of these proteins were found to have an uneven distribution across different vesicle subtypes, and their level may vary (Turiák et al., 2011), this concept of variable protein composition within the 100,000g emerged (Tauro et al., 2012; Tauro et al., 2013). Buck and colleagues made a remarkable discovery wherein nematode host cells took up exosomes secreted by *H. polygyrus*.

In addition to well-known RNA types like messenger RNAs (mRNAs), microRNAs (miRNAs), and ribosomal RNAs (rRNAs), EVs have revealed a diverse range of other RNA varieties. These encompass long and short noncoding RNA, tRNA fragments, piwi-interacting RNA, vault RNA, and Y RNA (Crescitelli et al., 2013; Huang et al., 2013; Ogawa et al., 2013; Cheng et al., 2014). Predominantly, the RNA molecules detected within EVs exhibit a length of approximately 200 nucleotides, although a smaller fraction extends to around 4 kb in size (Batagov and Kurochkin, 2013). Consequently, though some intact mRNAs and long non-coding RNAs are detectable to a certain degree, the prevailing proportion is likely fragmented in both exosomes and MVs (Abels and Breakefield, 2016). Interestingly, circular RNAs also exhibit significant abundance and stability within EVs (Li et al., 2015). When RNA becomes enveloped by the lipid bilayer membrane, it is believed to gain protection against degradation by RNase enzymes upon its discharge into the EVs milieu (Abels and Breakefield, 2016). Alternatively, a range of RNA types can form stable associations with ribonucleoproteins (RNPs), like argonaute 2 (AGO2), or with high-density lipoproteins (HDLs) and low-density lipoproteins (LDLs). Depending on the isolation technique used, these associations could relate to EVs or be incorporated into the EVs fraction (Arroyo et al., 2011; Vickers et al., 2011; Vickers and Remaley, 2012). The contents of helminth exosomes have primarily undergone investigation at the protein level, along with an examination of miRNA contents within these exosomes (Bernal et al., 2014; Buck et al., 2014). miRNAs constitute a highly conserved category of small, noncoding RNA molecules that orchestrate gene expression, exerting vital roles across developmental, developmental, physiological, and pathological processes (Chua et al., 2009; Manzano-Román and Siles-Lucas, 2012).

### 2.1 Lipid content of Evs

Lipids constitute a substantial portion of EV contents, albeit not as thoroughly understood as proteins and small RNAs. They are vital for vesicle organization creating membranes that protect and retain the internal cargo. Analysis of *H. polygyrus* EVs revealed an abundance of ether phospholipids called plasmalogens, believed to enhance membrane rigidity, thus improving resilience in the intestine (Drurey and Maizels, 2021). Plasmalogens are suggested to potentially shield against reactive oxygen species (ROS) or facilitate fusion with host membranes (Whitehead et al., 2020). Lipids themselves can exert biological activity, influencing cell uptake and directly modulating immunity (Drurey and Maizels, 2021). Lipids from *S. mansoni* have been found to trigger M2 polarization of macrophages (Assunção et al., 2017) and activate human eosinophils (Magalhães et al., 2018), with some proposing that EVs are the most effective delivery methods for these lipids.

Glycans represent another overlooked aspect of helminth EV biology. Both EV protein and lipids can undergo post-translational glycosylation, which is vital for regulating protein function (Varki, 2017), Glycans have been identified as critical determinants in EV uptake, influencing the cellular tropism of EVs (Williams et al., 2019). Characterization of *F. hepatic* EV glycans using lectin microarrays revealed distinctions from those found on the fluke’s tegument (Murphy et al., 2020). Eliminating external EV glycans with glycosidases hindered their uptake by a macrophage cell line, understanding the importance of these carbohydrates in the cellular uptake (de la Torre-Escudero et al., 2019). Glycans may also impact the distribution of helminth EVs treatment of mouse liver EVs with glycosidases resulting in their accumulation in the lung rather than the liver (Royo et al., 2019). EV glycans from *S. mansoni* resemble those found in total schistosomula extracts and include ligands for DC-SIGn 9CD209), the receptor on dendritic cells necessary for EV uptake (Kuipers et al., 2020), indicating the significance of EV glycosylation in interacting with host immune cells.

Additional proposed roles of glycans on EV surfaces include serving as a shield or source of decoy antigens and activating or evading host component pathways (Whitehead et al., 2020). Nevertheless, further research is required to fully grasp the functions of the subset of EV components. Novel proteomic techniques will aid in identifying glycosylation as well as other post-translational modifications such as phosphorylation and ubiquitination (Montaño et al., 2021).

### 2.2 Proteomics of parasitic-derived EVs

Several studies have investigated the protein composition of parasitic exosomes or exosome-like vesicles (Schorey et al., 2015), which have proven useful as diagnostic tools (Chen et al., 2020). Furthermore, exosomes generated by dendritic cells (DC) have been employed as vaccines to activate the immune response against certain types of cancer (Viaud et al., 2010). Exploring exosome and shed membrane vesicle protein content has been extensive since their initial discovery (Colombo et al., 2014). These exosomes exhibited a high abundance of proteins linked to exosome formation, including Alix, enolase, and HSP70, alongside proteins of undefined functions, miRNAs, and other non-coding RNAs (Buck et al., 2014). In the context of mapping host-parasite interaction and formulating novel anti-schistosome treatments, researchers have scrutinized excretory/secretory (ES) and tegumental proteins from various life stages of schistosomes (Loukas et al., 2007; Sotillo et al., 2015). EVs commonly contain proteins associated with processes like biogenesis, such as those associated with the endosomal pathway. Notably, components of the ESCRT complex including ALIX and TSG101, were noticeably enriched in the vesicle fraction (Abels and Breakefield, 2016). Moreover, proteins linked to the production and release of EVs, like RAB27A, RAB11B, and ARF6, were frequently identified (Abels and Breakefield, 2016). Furthermore, the repertoire of EVs encompassed several types of tetraspanins, including CD63, CD81, and CD9, along with proteins related to sight transduction (EGFR), antigen presentation (MHC I and MHC II), and other transmembrane proteins (LAMP1, TfR). Ordinarily, proteins linked to the endoplasmic reticulum, Golgi apparatus, and the cell nucleus were not detected within EVs (Théry et al., 2001). However, certain transcription factors like Notch and Wnt, which are typically found in the nucleus, were found within EVs (Kalra et al., 2012). Some studies have delved into the mechanism of loading proteins into vesicles and have suggested that this process involves the formation of oligomeric complex formation at the plasma membrane (Yang and Gould, 2013).

Neglected diseases often encompass helminth-borne illnesses, affecting approximately one-third of the global population with at least one helminth species infection (Hotez et al., 2008). The involvement of EVs in the initiation and progression of helminth infections has been observed in trematode species such as *E. Caproni*, *D. dendriticum*, and *F. hepatica*. The content of these EVs has unveiled the identity of previously uncharacterized proteins with excretory/secretory products (ESP), with approximately half of the proteins aligning with those identified in the secretome (Marcilla et al., 2012; Bernal et al., 2014). This might explain the aberrant protein secretion in flukes which is marked with the absence of secretion signals usually found in other parasitic groups. Metabolic enzymes like enolase, GAPDH, and aldolase, along with common exosome components like Hsp70 and annexins were identified among these proteins (Bernal et al., 2014). Moreover, variations in the composition of EVs were observed in *E. Caproni*, *D. dendriticum*, and *F. hepatica*, reflecting their distinct EVs secreted products. For instance, *E. Caproni* EVs lacked proteases, while *D. dendriticum* and *F. hepatica* EVs contained leucine aminopeptidase (LAP). Notable, *F. hepatica* EVs exhibited a significant presence of proteases, including cathepsins, which likely relate to its tissue migration pattern (Bernal et al., 2014). Besides proteins, helminth EVs were also found to contain miRNA, a discovery made for *D. dendriticum* EV (Bernal et al., 2014). Research on *S. japonicum*, *S. mansoni*, and *T. cruzi* revealed that these parasites may employ exosomes to release proteins into host cells (Bayer-Santos et al., 2013; Nowacki et al., 2015; Zhu L. et al., 2016). The demand for ATP, glycoconjugates, and ß-mannan is reliant on glycolysis and gluconeogenesis for site survival and virulence (Nawaz et al., 2019). Among the glycolytic pathway enzymes found in the parasites, vesicles were the multifunctional enzyme enolase (Marcilla et al., 2007). Various parasitic-derived EV proteins and their functions are summarized in **Table 1**.

**Table 1:** Summary of proteins identified in parasite derived EVs.

Parasites Names	Life Stage of Parasites	EVs Collection Protocol	Prominent Protein Identified	Total Number of proteins identified	Summary of functions and involved studies and discoveries	Reference
*S. japonicum*	Liver stage worm (28 days dpi)	The worm was harvested through mesenteric vein perfusion, washed in PBS, and maintained in RPMI-1640 medium supplemented with 100 U penicillin and 100 mg/ml streptomycin, and 10% exosome depleted fetal bovine serum at 37 °C with 5% CO_2_. EVs were collected by ultracentrifugation through a 3 K NMWL. The supernatant was filtered by using a 0.22 μm syringe filter and further exosome isolation kit was used for isolation.	Actin, Heat shock protein 70 (Hsp70), Glucose-6-phosphate (G6P), Heat shock protein 90 (Hsp90), Phosphoglycerate kinase (PGK), Rab-protein 11(Rab-11), Glutamine synthetase (GS), Rab-protein 10 (Rab-10)	403	Binding, catalytic activity, and translation regulation are the primary functions of this protein.	(Zhu L. et al., 2016)
*S. mansoni*	Adult worm (42 days dpi)	The worm was harvested through mesenteric vein perfusion, washed in PBS, and maintained in RPMI-1640 medium supplemented with 100 U penicillin and 100 mg/ml streptomycin, and 10% exosome depleted fetal bovine serum at 37 °C with 5% CO_2_. EVs were purified by differential centrifugation followed by filtration and sucrose density ultracentrifugation.	Heat shock proteins 70, integrins, glutathione-S-transferase (GST), tetraspanin (TSP-2), calpain, Enolase, 14–3-3 protein, Actin-2, Ubiquitin, Alpha tubulin, Rab 11, Histone (H3, H4, H2B)	130	Indications of vesicle-mediated release in these parasites, pivotal for host-parasite interplay, present a valuable resource for advancing vaccine and therapeutic strategies.	(Samoil et al., 2018)
*S. mansoni*	Adult worm (56 days dpi)	Adult worm was obtained by perfusion BALB/c mice and culture in serum free Basch medium at 37 °C, 5% CO_2._ EVs were purified using an Optiprep discontinuous gradient (ODG). The EVs were collected by ultracentrifugation.	Saposin B domain-containing protein, TSP-1, TSP-2, Sm29, Thioredoxin peroxidase, Annexin, Syntenin, Heat shock protein 70, Enolase, 14–3-3 protein, Calpain (Co2 family)	26	Adult *S. mansoni-secreted* EVs had an effective anti-schistosome effect and are potential vaccine and therapeutic candidates.	(Sotillo et al., 2016)
*S. mansoni*	Schistosomula	Cercaria were mechanically transformed into schistosomula and resuspended in culture medium containing DMEM at cultivated at 37 °C, 5% CO_2._ EVs were purified by differential centrifugation.	HSP-70, 14–3-3 protein homolog 1, Actine, Rab 11, 14–3-3 protein epsilon, CD63 antigen, Thioredoxin, Syntaxin 1a, Enolase, Calpain, Annexin, Serine protease inhibitors serpins	109	Employed in the study of the complicated biology of schistosome/host interactions.	(Nowacki et al., 2015)
*B. malayi*	(L3 stage)	Parasites were cultured in 50 mL RPMI 1640 at 37°C, 5% CO2. Differential centrifugation was used to isolate ELVs from 25 or 50 mL aliquots of Brugia culture media. The resulting supernatants were passed through 0.22 μm filters and ultracentrifugation were done to get ELVs.	Actin, HSP70, Beta-tubulin, Ubiquitin, Histone (H2B, H3, H4), Ras-related protein, 49s ribosomal protein (S16, S2, S3, S5, S9), 60S ribosomal protein (L10, L11, L3, L9)	32	Participating in an innovative mechanism through which parasitic nematodes of humans can actively steer host reactions during infection.	(Zamanian et al., 2015)
*T. muris*	Adult worms	Adult worms were harvested from the caecum of infected mice and washed in PBS containing 5X antibiotic/antimycotic and cultured in RPMI containing 1X AA, at 37°C, 5% CO2. For EVs isolation the media were obtained through differential centrifugation and the supernatant was ultracentrifuge for EVs isolation.	Peroxidasin, serpin B6, heat shock 90, actin-5C, Enolase,	148	By furnishing significant insights into whipworm biology, these findings, have played a role in shaping fresh approaches and targets for tackling nematode infection in both humans and animals.	(Eichenberger et al., 2018b)
*T. muris*	Adult worms	ES was collected by culturing adult parasites in RPMI media supplemented with 500 U/mL penicillin and 500 μg/mL streptomycin. Supernatants from worm cultures were collected and centrifuged. Supernatants were filtered using a 0.22 μm filter (Millipore) to remove cellular debris and microvesicles, and EVs were isolated by ultracentrifugation.	Tetraspanin, TSP- 1 domain-containing protein, Heat shock protein 70, Heat shock protein 90, Small heat shock protein, Enolase, Ras protein Rab, Apoptosis-linked gene 2 interacting protein X 1 (Alix)	125	EVs from *T. muris* have demonstrated the capacity to confer protection to mice in the face of subsequent *T. muris* infection. Moreover, these vaccinations amplified the antibody response to an excretory-secretory component that was devoid of EVs.	(Shears et al., 2018)
*T. circumcincta*	(L4 stage)	L4 were harvested and washed with PBS and cultured in nematode culture medium (RPMI-1640), containing penicillin/streptomycin, amphotericin B and gentamycin sulphate. The pooled ES products were filtered using a 0.22 μm syringes filter. Tci-L4ES samples were collected through ultracentrifugation.	Actin, Beta-tubulin, Keratin, Histones, Rab GTPase, Thioredoxin peroxidase, 60S ribosomal protein, Aspartic protease, metalloproteases,	85	The immunogenicity of the proteins within the EV-enriched fractions was affirmed by their interaction with IgA and IgG antibodies from sheep with prior infection.	(Tzelos et al., 2016)
*E. Caproni*	Adult worms	The *E. caproni* adult were harvested from ICR mice and the *F. hepatica* adults were obtained from cow livers were washed with PBS and maintained in RPMI-1640 culture medium containing 100 U penicillin and 100 mg/mL streptomycin. ELVs were purified by differential centrifugation and ultracentrifugation were done for EVs.	Actin, Beta-tubulin, Annexin A3, Enolase, GAPDH, Histone H3, Ubiquitin, Heat shock 10kDa protein 1, 14–3-3 protein zeta, Mucin-2	45	The occurrence of EVs elucidated the release of unconventional proteins in trematodes, and the indication of their internalization by host cells pointed towards a significant role of these structures in facilitating communication between the host and the parasite.	(Marcilla et al., 2012)
*T. pisiformis*	Metacestode larvae	Metacestodes collected from the peritoneum and greater omentum of rabbits were washed thoroughly in sterile 0.9% sodium chloride containing 100 μg/ml streptomycin and 100 IU/ ml penicillin. The larvae were again washed with RPMI-1640 and maintained in RPMI-1640 culture medium supplemented with 10% exosome depleted fetal bovine serum, 100 μg/ml streptomycin and 100 IU/ml penicil lin at 37°C under 5% CO2. ESP of *T. pisiformis* were purified by differential centrifugation and ultracentrifugation were done for EVs.	Heat shock cognate protein, Actin-1, rab, enolase, Calpain-A, Annexin, 14–3-3 protein, Vacuolar protein sorting-associated protein 4A, Ubiquitin	87	These reported proteins had immunomodulatory properties. It was linked to the interaction of cysticerci and their hosts.	(Wang et al., 2020)
*Opisthorchis felineus*	Adult worm	Adult parasites were recovered from bile ducts of the hamsters and washed 10 times in sterile saline. Production of extracellular vesicles was initiated on day 2, and the parasites were maintained in the RPMI 1640 medium supplemented with 1% of glucose, 100 units ml^−1^ penicillin G, 100 μg ml^−1^ streptomycin, and 0.25 μg ml^−1^ amphotericin B. EVs were purified by differential centrifugation and ultracentrifugation were done for EVs.	Ferritin, tetraspanin CD63, helminth defense molecule 1, globin 3, saposin B type domain-containing protein, 60S ribosomal protein, glutathione S-transferase (GST28), tubulin, and thioredoxin peroxidase	168	This finding elucidating the interaction between parasites and their hosts is shedding light on the roles of EVs released by parasites.	(Pakharukova et al., 2023)

### 2.3 miRNAs in helminths derived EVs

EVs have a diverse genetic material composition. DNA, including genomic and mitochondrial DNA, has been previously discovered (Guescini et al., 2010; Balaj et al., 2011; Waldenström et al., 2012). EVs were commonly found enriched in small RNAs, many of which were derived from ribosomal 18S and 28S rRNAs and tRNAs (Abels and Breakefield, 2016). An exploration focusing on the parasitic nematode *H. polygyrus* unveiled that miRNAs present in secreted EVs were assimilated by host cells. This uptake by target cells led to the regulation of numerous genes involved in the innate immune response against parasitic infections (Buck et al., 2014; Montaner et al., 2014). Notably, It has been demonstrated that miRNAs present in EVs originating from immune cells can incite and regulate innate immunity, yielding substantial and extensive impacts on the functionality and characteristics of immune cells (Mobergslien and Sioud, 2014). Moreover, miRNAs play pivotal roles in governing inflammatory processes (Rebane and Akdis, 2013). Significantly, miRNAs identified within helminth exosomes have been revealed to be dispatched to target cells, functioning to manage inflammation (Gracias and Katsikis, 2011; Bernal et al., 2014). A subset of those miRNAs (miR-125b and miR-31) seemed to share homology with counterparts found in human cells, where they played crucial roles in the function of T-cell subsets. These actions consequently governed immune responses concerning infections, inflammation, and autoimmunity (Siles-Lucas et al., 2015).

### 2.4 Functions of helminth derived miRNAs

The first miRNA discovered in nematodes, miRNA lethal 7 (let-7), has been isolated from the exosomes of several parasites, including *D. dendriticum* and *B. malayi* (Bernal et al., 2014; Zamanian et al., 2015). The role of let-7 in modulating the host’s immunological responses to the nematode *Toxocara canis* has been documented (Ma et al., 2016). Similarly, initially, Let-7 and Lin-4 were presumed to play roles in larval development and transition within *Caenorhabditis elegans* (Prucca et al., 2008). In another study, it was discovered that the transition of *S. mansoni* from miracidium to the sporocyst stage was notably reliant on the expression of let-7 during the miracidium stage (Xue et al., 2008). Additionally, the miRNA was identified within the EVs of *F. hepatica*, these EVs being associated with functions related to immune regulation (Cwiklinski et al., 2015). In line with another investigation, adult schistosomes secrete vesicles akin to exosomes that can be taken up by host immune cells found in peripheral blood. Subsequently, the miRNA cargo contained within these vesicles was implicated in the regulation of gene expression within the host (Liu et al., 2019). Additional functions of miRNAs uncovered within parasites encompass roles in embryogenesis, growth, and tissue development. miR-214 and miR-199, for instance, are implicated in TGF-b signaling and are found to be engaged in Schistosoma embryogenesis and development processes (Freitas et al., 2007; Okada et al., 2015). Correspondingly, miR-71b, miR-1, miR-36, and miR-124 have been identified as participants in the development progression of parasite embryos (Cai et al., 2013). Furthermore, miR-750 demonstrates specific enrichment in the EVs of female *S. japonicum*. This miR-750 demonstrates specific enrichment in the EVs of female *S. japonicum*. This miRNA exerts a noteworthy influence on regulating schistosome development and egg production. This assertion is supported by observations wherein the inhabitation of miR-750 led to a reduction in egg production within female schistosomes cultured *in vitro* (Du et al., 2020).

miRNAs play a significant role in regulating various physiological and pathological processes at the post-transcriptional level of gene expression, including organ development, immune responses, apoptosis, and cancer (Annese et al., 2020). Moreover, the identification of circulating miRNAs exhibiting robust stability in diverse host bodily fluids, including whole blood, serum, plasma, saliva, and urine captured the attention of researchers. This has ignited curiosity about the prospective utility of circulating miRNA in diagnosis and prognosticating infectious diseases (Raissi et al., 2021). Several circulating miRNA biomarkers have been employed in clinical applications in human diseases. However, this concept is only beginning to emerge in the field of infectious disease (Raissi et al., 2021). By repressing signaling pathways like Toll-like receptor and cytokine signaling, miR-155, miR-223, and miR-146 were effective in restraining hepatic damage during the later stages of microbial lipopolysaccharides infection (Taganov et al., 2006). The heightened presence of miR-1, miR-72, miR-87, miR-124, and let-7 within *T. spiralis* muscular larvae indicated their likely participation in parasite growth and metabolism processes (Chen et al., 2011). Additionally, another investigation documented the potential impact of miR-7 and Let-7 on the survival of *Echinococcus canadensis* within its intermediate host (Cucher et al., 2011; Fromm et al., 2013; Jin et al., 2013; Macchiaroli et al., 2015).

Likewise, previous studies have indicated that *S. mansoni* possesses the capability to release EVS abundant in small non-coding RNAs and proteins, a subset of which have been explored as potential vaccine candidates (Nowacki et al., 2015; Sotillo et al., 2016). Additionally, EVs derived from nematode parasites have been observed to overpower T helper type 2 immune responses in murine models. These vesicles contain miRNAs that effectively curtail the production of inflammatory cytokines by epithelial cells. These findings underscore the pivotal role played by miRNAs in immunoregulation and, correspondingly, highlight their potential utility as therapeutic agents and diagnostic tools (Buck et al., 2014). Another study concluded that the miRNAs in the EVs of *S. japonicum* play a potential role in its survival by regulating molecules in host cells, namely Pros1, Fam212b, and Clmp, which subsequent regulation of macrophage proliferation and TNF-α production (Liu et al., 2019).

Moreover, the role of miRNAs in conferring resistance to drugs in parasites has been unveiled. miRNAs possess the capability to modulate drug transporters, receptors, and ion channels, ultimately leading to decreased drug sensitivity. An illustrative instance is miR-1, which, through the regulation of unc-29 and unc-63 expression, curtails muscular responsiveness to levamisole and acetylcholine in *C. elegans* (Simon et al., 2008). Similarly, the developmental functions of lin-4 and let-7 have been clarified in *C. elegans* (Lee et al., 1993; Reinhart et al., 2000). Furthermore, miR-9551 expression has been identified in anthelminthic resistance strains of the parasites *H. contortus* and *T. circumcincta* (Sargison et al., 2007; Gillan et al., 2017). An exhaustive overview of proteins originating from diverse parasitic EVs, along with their respective functions, is presented in **Table 2**.

**Table 2:** Summary of miRNAs identified in parasite derived EVs.

Parasites Names	Prominent miRNAs	Total Nos of miRNAs identified	Function	Reference
*S. japonicum*	Sja-miR-36–3p, sja-miR-10–3p, sja-bantam, sja-miR-2a-3p, sja-miR-71a, sja-miR-3479–3p, sja-miR-2162–3p, sja-miR-71b-3p, sja-miR-2b-3p, sja-miR-61, sja-miR-277, sja-miR-307, sja-miR-2d-3p, novel-miR-7	14	It suggests that *S. japonicum* egg EVs have regulatory potential at the parasite-host interface	(Zhu S. et al., 2016)
*S. japonicum*	sja-miR-10–5p, sja-miR-125b, sja-miR-61, sja-miR-2b-5p, sja-bantam, sja-miR-2162–3p, sja-let-7, sja-miR-8185, sja-miR-277, sja-miR-36–3p, sja-miR-3489, sja-miR-2d-3p, sja-miR-3487, sja-miR-2c-5p, sja-miR-71a, novel-miR-2, novel-miR-6, novel-miR-8, novel-miR-22, novel-miR-26, novel-miR-15, novel-miR-23, novel-miR-24, novel-miR-30, novel-miR-12, novel-miR-19, novel-miR-5, novel-miR-16, novel-miR-18, novel-miR-3, novel-miR-10, novel-miR-20, novel-miR-25, novel-miR-9, Ocu-miR-191–5p	35	*S. japonicum* EVs may be involved in the pathogenesis of schistosomiasis through a mechanism involving the transfer of their cargo miRNAs to hosts	(Zhu L. et al., 2016)
*S. japonicum*	miR-125b, bantam, miR-61, miR-277b, miR-3505	2,569	The miRNAs with *S. japonicum* EVs possess the capability to influence host macrophages, showcasing the parasite’s ability to adjust the host’s immune response for its survival advantage. These findings offer a significant understanding of the dynamic interplay b/w schistosomes and their hosts, potentially facilitating the creation of innovative strategies for intervening in schistosomiasis.	(Liu et al., 2019)
*S. mansoni*	sma-miR-125b_R-1, sma-bantam, sma-miR-71a_R + 1, sma-miR-125a, sma-miR-36–3p, sma-miR-10–5p, sma-miR-61_R + 1, sma-miR-2a-3p_R-1, sme-lin-4–5p_, sja-miR-2162–3p, sja-miR-277_R + 2, PC-5p-12974_124, sja-miR-277_R + 1, PC-3p-8606_176, PC-5p-1634_720, sme-mir-749-p5, sma-let-7, sma-miR-71b-5p, PC-5p-15294_107, PC-3p-8590_176, PC-5p-10382_150, sma-miR-3479–3p, PC-5p-14055_115, PC-5p-1776_672, sja-miR-3492	25	Exosomes derived from schistosomes might show significant roles in host-parasite interactions and could be used to develop vaccines and therapeutics	(Samoil et al., 2018)
*S. mansoni*	Sma-mir-61, sma-mir-36a, sma-mir-71a, sma-mir-2162, sms-mir-190, sms-bantam, sma-miR-1a, sma-mir-71b, sma-mir-n1, sja-miR-277, sma-mir-96, sma-mir-2a, sme-miR-315–5p, sma-miR-36b, sme-miR-71c-5p, sma-mir-10, sma-mir-125c, sma-mir-125b, sma-mir-3479, sma-miR-1b, sja-miR-7–5p, sma-mir-745, sja-miR-3499, sma-miR-3492, sma-mir-8, sma-mir-2c, egr-miR-87–3p, sma-mir-76, sma-mir-281, sja-miR-219–5p, sme-miR-61a-3p, emu-miR-745, sja-miR-3488, sma-mir-2149	205	The identification of *S. mansoni* EVs and the comprehensive analysis of their cargo, which includes both proteins and non-coding RNAs (sncRNAs), introduces a significant and novel element to the intricate dynamics governing schistosome/host interactions.	(Nowacki et al., 2015)
*H. polygyrus*	miR-83/29, miR-60, miR-63/425, miR-79/9, miR-239, miR-7, miR-77, miR-240/193, miR-57/10, miR-8/200, lin-4/125, miR-5884, miR-44, miR-100, Bantam, miR-71, mir-263, miR-87	263	Helminths endeavor to exert an influence on their hosts, offering a mechanistic basis for the transfer of RNA between different species	(Buck et al., 2014)
*B. malayi* (L3 stage)	Bma-let-7, bma-miR-1, bma-miR-9, bma-miR-92, and bma-miR-100b	21	The discoveries suggest an innovative mechanism through which parasitic nematodes that infect humans can effectively steer host responses during infections.	(Zamanian et al., 2015)
*T. muris*	let-7, miR-2, miR-9, miR-34, miR-36, miR-44, miR-60, miR-72, miR-81, miR-86, miR-87, miR-92, miR-228, miR-236 and miR-252	56	By furnishing vital insight into whipworm biology, these findings contribute to the formation of fresh strategies and targets for tackling nematode infections in both humans and animals	(Eichenberger et al., 2018b)
*D. dendriticum*	let-7, miR-10, miR-2b, bantam, miR-2e, miR-71, miR-190, miR-7, miR-125	20	These vesicles can encapsulate particular RNAs, ensuring durability and safeguarding against RNA degradation in bodily fluids. This encapsulation also offers a mechanism for managing interactions between hosts and parasites. The present results should establish a robust groundwork for devising innovative approaches to manage this non-model organism and its associated parasites.	(Bernal et al., 2014)
*T. pisiformis*	miR-71–5p, miR-10a-5p, miR-let-7–5p, miR-745–3p, miR-219–5p, miR-124–3p, miR-4989–3p, novel-mir-3, novel-mir-7, novel-mir-8, novel-mir-11	41	MiRNAs contained within *C. piriformis* exosome-like vesicles have immunomodulatory properties. It is also important in the interaction of cysticerci and their hosts	(Wang et al., 2020)

## 3 The function of helminth derived EVs

**Figure 2:**
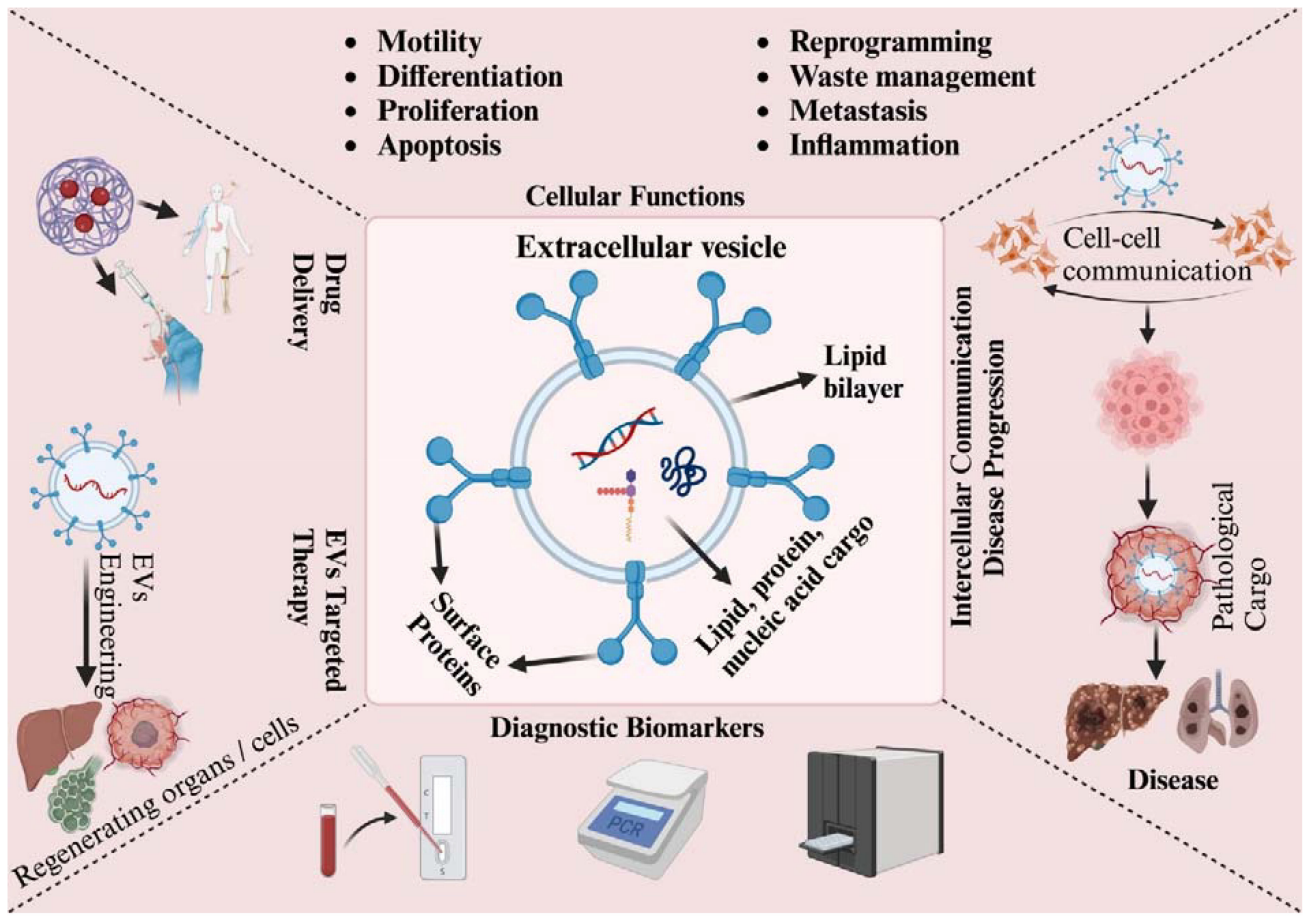
Schematic presentation of EVs’ role in cellular, pathological, and clinical applications. EVs enable communication through their surface proteins and cargo, which comprise proteins, lipids, and nucleic acids. They participate in various biological processes, including cell motility, differentiation, proliferation, apoptosis, reprogramming, waste management, metastasis, and inflammation. EVs are linked to their clinical potential in several areas: they can be used for diagnosis due to their biomarker properties, drug delivery owing to their targeting capabilities, and targeted therapy by harnessing their communication abilities. EVs contain bioactive cargo that influences the fate of target cells. To improve their functionality and specificity, EVs can be engineered with surface modifications or by loading them with specific therapeutic cargo. However, EVs released under pathological conditions, such as from cancer or infected cells, can contribute to the spread and progression of diseases.

Research focusing on EVs has unveiled their role in governing various cellular functions such as motility, polarization, immune responses, and development processes. Additionally, these vesicles have implications for the progression of diseases such as cancer and neurodegeneration (Zhang and Grizzle, 2011; Colombo et al., 2014; Abels and Breakefield, 2016; Riaz and Cheng, 2017). The mounting body of evidence indicates that both EVs and their cargo of circulating exosomes, particularly the miRNA content, hold promise as potential biomarkers for specific diseases (Li et al., 2015). Helminths contain a wealth of novel molecules that could potentially catalyze advancements across multiple domains of biomedicine. Previous literature concerning host-parasite interaction has yielded noteworthy outcomes, particularly in the identification of possible new targets for diagnosis, treatment, and vaccine development (Hotez et al., 2008; Toledo et al., 2011) (as shown in **Figure 2**).

### 3.1 Helminth-derived EVs role in intercellular communication

EVs the magic bullets have prompted researchers to investigate the role in the pathogenicity, invasion, and persistence of parasitic infections (Marcilla et al., 2012). Currently, EVs are recognized as key players in the interaction between hosts and parasites (Zakeri et al., 2018), facilitating communication between cells. during infection, the immune system is continuously engaged with various products from helminths, including EVs, which can either stimulate or regulate the immune response (Zheng et al., 2016). For instance, recent studies have shown that parasite EVs can influence the activation of macrophages and modulate inflammatory reactions (Nazimek et al., 2016). The transfer of EV-associated RNAs, such as microRNAs, between cells is emerging as an important mechanism for inducing changes in gene expression through epigenetic modification and post-transcriptional regulation (Zakeri et al., 2018). The uptake of helminth EVs by host cells underscores the substantial role these vesicles play in enabling communication between the parasite and its host (Marcilla et al., 2012). Initial investigations have indicated that EVs produced by a nematode like *H. polygyrus* can modulate inflammatory reactions in both cultured cells and a mouse model. These observations provide insights into the potential mechanisms through which these nematode-derived EVs might facilitate inter-phylum communication and potentially contribute to dampening the host’s inflammatory response (Coakley, 2014).

### 3.2 EVs role as immunomodulators

Helminthiasis is a parasitic disease that exhibits a significant prevalence owing to its capacity to manipulate the host’s immune system, leading to the inhibition of parasite expulsion. Helminths offer a unique opportunity to investigate how parasites adjust the host’s immune mechanisms to mitigate the negative effects of parasitic infestation. Interestingly, the removal of helminths can sometimes lead to even more harmful consequences. The immune reactions triggered by helminths involved a blend of innate defense and a TH2 response. This combined response works to incapacitate, break down, and dislodge the parasites (Maizels et al., 2012b). Notably, helminth infections are characterized by the dominance of TH2-related immune reactions and the inhibition of the Th1/Th17 immune response, enabling the parasite to endure within a “modified Th2 environment” (Hewitson et al., 2009; McSorley and Maizels, 2012). Understanding the Immune regulation orchestrated by helminths could potentially open new avenues for addressing immune disorders allergies, autoimmunity conditions, and inflammatory bowel disease (McSorley and Maizels, 2012; Zaccone and Cooke, 2013). Numerous clinical trials have been conducted to explore the potential helminths for treating autoimmune diseases (Wolff and Broadhurst, 2012). Additionally, there have been reports of enhanced acceptance of allograft (transplant of organs or tissues from a different individual of the same species) in various species with helminth infection. This suggests that helminth infection or specific products derived from immune-modulating helminths might hold promise for future transplantation procedures (Johnston et al., 2014). Consequently, the scientific investigation has honed in on the “excretory-secretory” products released by live parasites. These products have the remarkable ability to interfere with various aspects of the host’s immune system, ranging from initial recognition to the final stage of immune responses (Hewitson et al., 2009). Notably, certain proteins by Schistosome namely alpha-1 and omega-1, play key roles in this modulation. Alpha-1, released from schistosome eggs, prompts the release of IL-4 and the discharge of IL-4 and reduces the activity of basophils in both human and mouse systems. This initiates an environment that favors the TH2 immune (Hewitson et al., 2009). Omega-1, a ribonuclease abundantly released by schistosome eggs, is thought to trigger the immune reactions necessary for facilitating the passage of eggs through the host’s tissues, aiding their reaction. Recent evidence supports the idea that omega-1 can directly initiate Th2 immune responses further confirming this hypothesis (Thomas et al., 2003; Hewitson et al., 2009).

A study on antigens released from the tegument of *F. hepatica* found that it managed to impede mast cells, which usually perform a crucial function in microbial infections. These modulatory changes resulted in suppressing cytokine signaling which had in turn affected the self-regulatory Th1-dependent inflammatory processes (Vukman et al., 2013). Another study found that ESP molecules released by *F. hepatica* can direct the immune response toward a more beneficial but non-protective Th2-mediated environment (Dalton et al., 2013). Cathepsin L, peroxiredoxins, and helminth defense molecules like HDM-1/MF6p are examples of immunomodulatory molecules. These molecules have the potential to treat autoimmune disorders and chronic inflammation in both humans and animals (Dalton et al., 2013). Notably, two of these proteins, peroxiredoxins and cathepsins, have been isolated from *F. hepatica* EVs (Marcilla et al., 2012). The third immunomodulatory molecule, HDM-1/MF6p has been found in exosomes from a related trematode species called *D. dendriticum* (Bernal et al., 2014), as well as *F. hepatica* and *Echinostoma caproni* (Montaner et al., 2014). HDM-1/MF6p, derived from *F. hepatica*, has biochemical and functional properties like peptides found in the human immune system, bearing a striking resemblance to CAP18. *F. hepatica* HDM-1 was found to modulate the activation of innate immune cells in response to classical Toll-like receptor (TLR) ligands such as lipopolysaccharide (LPS). This highlights its potential as a promising therapeutic candidate in autoimmune disease (Robinson et al., 2011). Furthermore, HDM-1 demonstrates its ability to inhibit the inflammatory response of macrophages induced by LPS, effectively suppressing the production of TNF and IL-1. In mouse experiments, those given a single dose of FhHDM-1/MF6p either before or after exposure to bacterial LPS showed significant reductions in circulating levels of TNF and IL-1 (Robinson et al., 2011; Robinson et al., 2012; Dalton et al., 2013). A recent discovery has unveiled the functional aspect of FhHDM-1/MF6p as a protein that binds to heme, suggesting its potential role as a heme chaperone. This role may be significant in overseeing essential physiological activities for the parasites, including the management of heme trafficking and storage. It’s important to note that this protein doesn’t appear to act as the primary ligand for LPS (Martínez-Sernández et al., 2014). In the context of disease prevention, *F. hepatica* ESP has demonstrated its ability to thwart the onset of type 1 diabetes (T1D) in non-obese diabetic (NOD) mice. This effect was linked to the suppression of interferon (IFN) secretion from auto-reactive T cells and a shift towards producing IgG1 auto-antibodies (Lund et al., 2014). Furthermore, recent investigations have reaffirmed the immunomodulatory impact of *H. polygyrus* EVs in a murine model (Coakley, 2014), corroborating earlier findings with ESP from the same nematode (Maizels et al., 2012a).

### 3.3 EVs role in the delivery system

The exploration of exosomes as potential therapeutic tools is a relatively recent development. Exosomes are increasingly being recognized as a novel avenue for therapeutic applications, serving as a transport system and cell-free tool within the realm of regenerative medicine (Fleury et al., 2014). The role of exosomes as conveyors of biological cargo can be harnessed for the efficient delivery of therapeutic compounds. These vesicles can be employed as carriers for therapeutic RNAs, leveraging their capacity to modify their RNA content either by transferring specific miRNAs back to the parent cell or by directly transporting miRNAs within the vesicles. The feasibility of this approach has been demonstrated through the utilization of EVs derived from tumors to deliver therapeutic small-interfering RNAs (Robbins and Morelli, 2014).

However, certain aspects related to utilizing exosome-like particles to deliver miRNAs for therapeutic intentions are still under scrutiny. These encompass challenges such as precise cell targeting, cargo stability, and the precise impact of distinct miRNAs on gene expression within target cells. Notably, exosomes generated in laboratory settings by cells transfected with miRNAs have demonstrated adeptness in effectively transporting miRNAs to designated target cells. This achievement is facilitated by manipulating the donor cells, such as dendritic cells, to express specific molecules that interact with receptors on the surface of the target cells. This interaction leads to the fusion of exosomes with distinct components of their membrane, contributing to a successful and targeted delivery (Siles-Lucas et al., 2015). In certain instances, the need for such hybrid exosome engineering can be circumvented due to the presence of distinct exosome subgroups that exhibit specific preferences for cells. A noteworthy example lies in the occurrence of antigen-driven unidirectional miRNA transfer from T cells to antigen-presenting cells (APC), facilitated by the transmission of CD63+ exosomes during the formation of the immune synapse (Mittelbrunn et al., 2011). To effectively target specific cells and elicit the desired effects, a comprehensive grasp of various factors is essential. This includes comprehending the specificity of tropism with diverse exosome subpopulations, the efficacy of strategies employed to manipulate exosomes, and the preservation of the integrity of transfected miRNAs before their encapsulation within exosomes by the donor cell (Akao et al., 2011). The direct incorporation of miRNAs into isolated exosomes can serve as an alternative to transfecting cells with miRNAs. Subsequently, exosomes can serve as carriers for gene delivery, effectively substituting viral particles and circumventing associated adverse effects. This is achievable because exosomes derived from a patient’s source (such as plasma) can feasibly function as non-immunogenic gene delivery vectors, especially for that individual (Wahlgren et al., 2012). Exosomes can be loaded with miRNA using methods like chemical transfection and electroporation, with multiple kits offered by different companies. However, electroporation affects the exosome colloidal stability (Hood et al., 2014), and electroporation can cause siRNA precipitation thereby lowering the efficiency with which siRNA is loaded into the extracellular vesicles (Kooijmans et al., 2013). Nonetheless, electroporation appears to be more efficient than transfection (Wahlgren et al., 2012), and in any way, better and/or alternative methods for loading exosomes should be developed.

The primary challenge in utilizing natural exosomes stems from their production of a diverse array of nano-sized vesicles, the composition of which varies based on the cell type and activation state. Additionally, the methods for effectively isolating and capturing these vesicles in a discernible manner are intricate, and these complexities hinder their application for larger-scale delivery of constituents to specific target cells. Generating artificial nanovesicles has been suggested as a more well-organized way that forces cells through a hydrophilic (Jo et al., 2014). Certain research endeavors have delved into the feasibility of artificially engineering exosome-like particles. Given the structural similarity of the exosome membrane’s bilayer to that of a liposome, liposome-based systems stand out as promising candidates for creating exosome mimics. This approach could potentially streamline the production of exosome-like particles, and the straightforward nature of using well-characterized components might enhance the feasibility of such systems.

Nonetheless, the essential exosomal components necessary for constructing functional and targeted exosome mimetics remain to be identified (Kooijmans et al., 2013). However, several challenges need to be addressed before contemplating the utilization of miRNA cargo for human applications. For instance, individual miRNAs have the potential to influence a broad spectrum of genes, thereby intensifying the complexity of achieving precise cell and gene targeting. This represents one of the more intricate hurdles that must be overcome (Pawitan, 2009).

### 3.4 EVs role as a novel vaccine

Helminth parasites remain among the most neglected tropical diseases. Currently, there are no effective vaccines for humans and only a few for animals (Hewitson and Maizels, 2014). In addition to their role in pathogenesis and immunomodulation, EVs from helminths contain some vaccine-candidate antigens, which can be present in the cargo and membrane of EVs (Mekonnen et al., 2018). The use of EVs as a vaccine is gaining interest due to their immunomodulatory role and ability to generate specific antibodies (Coakley et al., 2017). In various helminths, tegumental tetraspanins (TSPs) are essential for forming the tegument, the outer membrane that directly interfaces with host tissues (Piratae et al., 2012). The large extracellular loops (LELs) of TSPs have shown effectiveness as vaccine antigens in numerous helminth infection models. For example, mice immunized with the LELs of two *S. mansoni* TSPs (Sm-TSPs-1 and Sm-TSP-2) experienced a notable reduction in adult worms and liver egg counts post-infection (Tran et al., 2006). Similarly, mice vaccinated with a fusion of the Sj23 TSP and other vaccine candidates exhibited a significant decrease in worm burden and liver egg counts following an *S. japonicum* challenge (Zhu et al., 2012).

Saposins, activators of sphingolipid hydrolases, have also been evaluated in helminth vaccine trials (Mekonnen et al., 2018). In one study, rabbits immunized with a recombinant Saposins-like protein showed substantial reductions in adult worm burdens and egg loads in feces and bile after *F. hepatic* infection (Espino and Hillyer, 2004). Saposins domain-containing proteins have been identified in the EVs of several helminths, including *S. mansoni* and *F. hepatica* where they are the most abundant proteins (Sotillo et al., 2016). Paramyosin, found on the surface of schistosomula (McManus et al., 2020), the tegument, and acetabular glands is considered a promising vaccine against *S. japonicum* (Karmakar et al., 2014). Immunizing pigs with recombinant Paramyosin resulted in a 33–34% reduction in adult worm burden (Chen et al., 2000), while mice vaccinated with parasite-derived Paramyosin can cause a 62–86% reduction (Mekonnen et al., 2018). This protein has also been detected in the EVs of *O. viverrini* (Chaiyadet et al., 2015). Leucine aminopeptidases, and metallopeptidase that remove N-terminal residue from protein and peptidase (Matsui et al., 2006), have demonstrated vaccine efficacy against *F. hepatica* in animal models (Mekonnen et al., 2018). Sheep immunized with both native and recombinant leucine aminopeptidase showed a 49–89% reduction in adult worm burden after *F. hepatica* challenges (Maggioli et al., 2011). This enzyme is abundant in the EVs of both *S. mansoni* and *F. hepatica* (Sotillo et al., 2016).

Cathepsin L, a protease found in the EVs of *F. hepatica* and *B. malayi* (Cwiklinski et al., 2015), has been shown potential as a vaccine antigen. Immunizing cattle and sheep with parasite-derived cathepsin L led to a 42–69% and 34% reduction in adult worm burden respectively (Mekonnen et al., 2018). Another study found that cattle vaccinated with recombinant cathepsin L had a 48% reduction in adult worm burden (Golden et al., 2010). Additionally, mice immunized with recombinant cathepsin B showed reductions of 59%, 56%, and 54% in adults’ worm, liver egg, and intestinal egg burden respectively, after an *S. mansoni* challenge (Ricciardi et al., 2015). Cathepsin B has also been detected in the EVs of *F. hepatica* and *S. japonicum* (Cwiklinski et al., 2015).

The subcutaneous injection of EVs purified from *E. caproni* could reduce symptom severity and mortality in mice after experimental infection (Trelis et al., 2016). Similarly, EV vaccination in mice produced specific antibodies, providing protective immunity against *H. polygyrus* (Coakley et al., 2017). Additionally, EVs derived from *T. muris* have been found to offer protection against subsequent infections, with several proteins identified in these EVs proposed as potential vaccine candidates. However, further research is needed to explore the potential of parasite-derived EVs as new vaccine candidates (Eichenberger et al., 2018a).

It is also discovered that 31% of the proteins identified in EVs secreted by *S. mansoni* are homologous to the previously described vaccine candidates, with many of these proteins present throughout the parasite’s lifecycle. This indicates that an EV-based vaccine could target different life stages of the parasite and be effective against *S. mansoni* infection (Sotillo et al., 2016). Despite their biological complexity and the limited understanding of their specific mechanisms and interactions with the immune system, EVs from *H. polygyrus* have been shown to suppress macrophage activation and target the IL-33 pathway (Coakley et al., 2017), which has significant potential for future vaccine development, paving the way for new treatment options against parasite infection (Mekonnen et al., 2018).

### 3.5 EVs as possible biomarkers: potential for early diagnosis

The development of high-throughput assays, now including EV arrays (Jørgensen et al., 2013), has greatly enhanced our understanding of the interaction between host and parasites, particularly the pathways through which EVs influence host immune cells. Proteomics analysis focusing on the protein concentrated in EVs has yielded crucial data for identifying and isolating key components that can be used as biomarker diagnostic tools, and for vaccine development (Zakeri et al., 2018). EVs are known to contain a diverse array of molecules and are protected by a membrane, ensuring their stability over time (Carrière et al., 2016). They are distributed in various body fluids and organs, making them attractive for biomarker-based diagnosis (Zakeri et al., 2018). EVs have been extensively studied as potential biomarkers in cancer, where the contents of exosomes differ from healthy individuals (Carrière et al., 2016). Specific miRNA signatures in exosomes have been associated with certain cancer types, offering a novel approach for diagnosis and treatment monitoring (Tokuhisa et al., 2015). In infectious diseases, EVs isolated from serum samples have shown diagnostic potential (Singh et al., 2012). However, the physical location of the pathogen can impact the effectiveness of this method or the appropriate method for sample collection (Zakeri et al., 2018). Various components of EVs, such as miRNAs and proteins, have been identified in different trematode species and hold potential as additional biomarkers for detecting fluke infections. Specifically, miRNAs of helminth origin found in serum EVs could serve as biomarker candidates for the diagnosis of Schistosome infections. Previously researchers have confirmed the diagnostic potential of four schistosomal miRNAs (Bantam, miR-2c-3p, miR-3488, and miR-2a-5p) in serum EVs with the first miRNAs exhibiting an AUC greater than 0.91. Notably, two of these EV-derived miRNAs, Bantam and miR-2c-3p, showed sensitivity and specificity rates of 85.71%/94.12% and 85%/93.75%, respectively (Mu et al., 2020).

The cathepsin B1 from *O. viverrini* has emerged as a potential biomarker candidate in sera (Sripa et al., 2012). Overall, the release of EVs appears to facilitate a mutually beneficial interaction between hosts and helminths. Highly immunogenic components in EVs suggest an effective immune response against them, offering potential biomarker candidates. Conversely, helminths continuously evolve strategies to evade host immunity and establish infections, and the production of EVs containing diverse materials may be a key tactic in this process (Zakeri et al., 2018). Various extracellular vesicles (EVs) components, such as miRNAs and proteins, have been identified across different trematode species, presenting potential biomarkers for detecting fluke infections (Mekonnen et al., 2020; Ovchinnikov et al., 2020). Studies on EVs released by *F. hepatica*, a major cause of human fascioliasis and an emerging zoonotic pathogen, identified the diagnostic antigen cathepsin L1 (Sarkari and Khabisi, 2017). Liver flukes are suggested to secrete EVs into host bile, indicating that the molecular content within EVs could potentially be exploited for diagnosing fascioliasis (Zakeri et al., 2018). Tapeworm-derived miRNAs can be consistently detected in the blood serum or plasma of mammalian hosts during infection, despite these parasites not residing within the host’s bloodstream (Guo and Zheng, 2017). EV compositions, including small RNA and protein profiles, have been considered for certain cestode species (Wang et al., 2020b). In *E. multilocularis* 18 miRNAs were identified from metacestode EVs. Among these, the four most abundantly expressed miRNAs (emu-mir-71–5p, let-7–5p, miR-4989–5p, and miR-10–5p), as well as emu-miR-2c-3p, were detectable in the sera of parasite-infected mice (Ding et al., 2019). Notably, a threonine tRNA derived small sequence from the 5^/^end had a dominant read count higher than the combined read counts of all 18 miRNAs. This indicates that small RNAs, such as tsRNAs, could be more effective diagnostic biomarkers than miRNAs in EVs (Mu et al., 2020). Studies on *E. granulosus* have examined EVs isolated from hydatid cyst fluid (HCF) directly from hosts with cystic echinococcosis (CE) (Zhou et al., 2019), and cultured protoscoleces (Nicolao et al., 2019), leading to the identification of highly immunogenic antigens like antigen 5, Antigen B, P29, and endophilin-1. However, the following studies have shown that these miRNA levels in human blood are too low for reliable use as biomarkers for infection detection or treatment monitoring, even with locked nucleic acids (LNA)-based RT-qPCR analysis (Macfarlane et al., 2020). Studies have also investigated the composition of nematode-derived extracellular vesicles as potential diagnostic tools for blood-dwelling and blood-feeding nematodes (Mu et al., 2020). For instance, EVs released from *Brugia malayi microfilariae* were found to contain 576 proteins and a unique miRNA profile with potential diagnostic markers (Ricciardi et al., 2021). Additionally, analysis of EVs from *N. brasiliensis*, a model for human hookworm infection, revealed numerous proteins and miRNAs that could serve as promising diagnostic candidates (Eichenberger et al., 2018a).

## 4 Conclusion

Recently, there has been a notable increase in research focusing on the biological attributes of exosomes. In the context of host-parasite interactions, parasite exosomes play a pivotal role, transferring molecules such as immunomodulatory compounds, and nucleic acids between parasites and host cells. Proteomic and transcriptomic analyses of parasite-derived exosomes have revealed numerous proteins and miRNAs essential for parasite reproduction, survival, and immune system modulation. These exosomal constituents are specific to each parasite species and its host. Understanding the functional roles of these proteins and miRNAs holds promise for uncovering new communication pathways between parasites and hosts via parasite exosomes, their roles in immune responses, their roles in immune responses, and their potential applications in diagnosing and treating parasitic diseases. The connection between EVs and infection biology is significant, opening pathways for developing new therapeutic strategies, biomarkers, and vaccines in various fields.

## 5 Future perspective

The investigation of EVs in zoonotic helminth biology presents an exciting frontier with significant implications for diagnostics, therapeutic development, and molecular delivery systems. As research advances, several key areas warrant focus to fully leverage the potential of EVs in addressing helminth infections. Further research is needed to explore the potential of helminthic EVs to deliver genetic material, such as RNA interference (RNAi) molecules, to modulate gene expression in both the parasite and the host could open new therapeutic avenues. Engineering synthetic EVs that mimic the natural properties of helminth derived EVs could offer a versatile platform for drug delivery and therapeutic applications, tailored to specific needs and conditions. Investigating how environmental changes, particularly climate change, affect the production and function of helminth derived EVs could provide insights into how these factors influence disease transmission and emergence. Using EVs as indicators of ecosystem health and the presence of zoonotic helminths in various environments could enhance our ability to predict and manage outbreaks. Collaboration among parasitologists, immunologists, ecologists, and public health professionals is essential to integrate findings from EV research into comprehensive strategies for managing helminth infections within the One Health framework. Incorporating advanced technologies such as CRISPR/Cas9, high-throughput sequencing, and bioinformatics will accelerate the discovery and application of EV-based diagnostics and therapies. The future of EV research in zoonotic helminth biology holds immense potential to transform our approach to diagnosing, treating, and managing helminth infections. By focusing on the unique properties of EVs, we can develop innovative solutions that address both the clinical and ecological challenges posed by these widespread parasites.

## Author contributions

AQ: Writing – original draft. AW: Writing – original draft. HR: Writing – review & editing. SN: Writing – original draft. SK: Writing – review & editing. SR: Writing – review & editing. KA: Writing – review & editing. MK: Writing – review & editing. MA: Writing – original draft. HU: Writing – original draft. SS: Writing – original draft. ZX: Writing – review & editing, Validation, Funding acquisition. MZ: Writing – review & editing.
